# Cooked Black Turtle Beans Ameliorate Insulin Resistance and Restore Gut Microbiota in C57BL/6J Mice on High-Fat Diets

**DOI:** 10.3390/foods10081691

**Published:** 2021-07-22

**Authors:** Yuqing Tan, Christina C. Tam, Shi Meng, Yan Zhang, Priscila Alves, Wallace Yokoyama

**Affiliations:** 1 Beijing Laboratory for Food Quality and Safety, College of Food Science and Nutritional Engineering, China Agricultural University, Beijing 100083, China; yuqingtan@cau.edu.cn; 2 Healthy Processed Foods Research Unit, Agricultural Research Service, United States Department of Agricultural, Albany, CA 94710, USA; priscila.alves@usda.gov (P.A.); wally.yokoyama@usda.gov (W.Y.); 3 Foodborne Toxins Detection and Prevention Research Unit, Agricultural Research Service, United States Department of Agriculture, Albany, CA 94710, USA; christina.tam@usda.gov; 4 Nestlé R & D (China) Ltd., Beijing 100015, China; 5 Key Research Laboratory of Agro-Products Processing, Institute of Food Science and Technology, Chinese Academy of Agricultural Sciences, Beijing 100193, China; 6 Experimental Seafood Processing Laboratory, Costal Research and Extension Center, Mississippi State University, Starkville, MS 39567, USA; yzhang@fsnhp.msstate.edu

**Keywords:** cooked black turtle bean, insulin resistance, LDL, gut microbiome

## Abstract

Colored common beans are associated with health promoting and chronic disease prevention effects. Male C57BL/6J mice were fed high-fat (HF) diets supplemented with cooked black turtle beans (HFB) to prevent obesity related insulin resistance. Mice on both HF and HFB were obese compared to mice fed a low-fat (LF) diet. Plasma low density lipoprotein (LDL) and triglyceride concentrations of mice fed HFB diet were 28% and 36.6% lower than those on HF diet. Homeostatic model assessment of insulin resistance (HOMA-IR) index of mice fed HFB diet was 87% lower than that of mice fed HF diet. Diabetes related biomarkers, gastric inhibitory polypeptide (GIP), leptin, glucagon, and inflammatory cytokines interleukin 4 (IL-4) and IL-5, 10 and 12, IFN-g and TNF-α were significantly affected by HFB diet. Pparα, Cyp7a1 and Fasn were down-regulated by HFB diet while *LDL-R, Srebp-2*, *Adipoq* and *Slc2a4* were up-regulated by HFB diet. The ratio of Firmicutes/Bacteroidetes (F/B) was also decreased 64.1% by HFB diet compared to HF diet. The results indicated that cooked black turtle bean consumption could ameliorate insulin resistance and lower plasma LDL in mice fed HF diet through glucose signaling pathway and JNK/c-Jun pathway. Meanwhile, cooked black turtle bean consumption restored the gut microbiome.

## 1. Introduction

Legumes and their products play an important role in traditional diets in many regions such as Europe, Mediterranean, Asia, South America and North America [1]. In the United States, approximately 5.7 pounds of dry beans were consumed per capita in 2017 [2]. Varieties of Phaseolus vulgaris (navy bean, pinto bean, kidney bean and black turtle bean) are the major beans consumed in US [2]. Their importance as a protein source is exemplified by the US Department of Agriculture’s Food Guide Pyramid that lists legumes in the same high protein group as meat, poultry, fish and egg [3]. In recent years, colored common beans such as black turtle bean, black soybean, lentil, kidney bean and pinto bean have attracted more consumer interest because their darker colors are associated with health promoting and anti-chronic disease properties [4,5]. Among these commonly consumed beans, black turtle bean possesses the highest phenolic content (6.89 mg GAE/g) and antioxidant activities (79.27 μmol TE/g) [6]. Tan et al. [7] not only reported extremely high phenolic content (599.22 mg GAE/g) and antioxidant capacity (35,830 μmol TE/g) of purified and fractionated phenolic extracts from black soybean and black turtle bean, but also found that some phenolic fractions possessed inhibitory activities against lipase, α-glucosidase and α-amylase, which were even higher than commercial drugs used for diabetes treatment [8]. Most in vivo studies of common bean (P. vulgaris) varieties have been conducted with rats that had diabetes induced by specific drugs. Hernandez-Saavedra et al. [9] reported the protective effect of cooked common beans on β-cell damage in STZ-induced diabetic rats. Monk et al. [10] reported the mitigation of colitis and associated inflammation in C57BL/6J mice fed AIN93 diet containing 20% cranberry bean flour. Most studies focused on health-promoting effects of phenolic compounds but many phenolics are well-known to have poor bioavailability and require microbial metabolism to transform them into smaller bioactive compounds [11]. Legume was usually consumed whole while phenolic compounds are concentrated in the seed coat. Therefore, to investigate the health-promoting effect of consumption of the whole legume seed research is badly needed.

The health benefits of legumes have advanced the development of new foods which contain beans, such as chips, pasta, breads, as well as interest in traditional foods because of their high content of proteins, fiber and minerals. However, legumes are consumed after heat processing and their digestibility is significantly affected by heating processes [12]. The anti-diabetic and anti-inflammatory effects of diets enriched with cooked whole common beans such as black turtle bean have not been well studied. In addition to their antioxidant activity and digestive enzyme inhibitory capacities, legumes contain macronutrients such as resistant starch, non-starch polysaccharides, and proteins that are resistant to digestion and may retard the postprandial glycemic response [13,14,15]. These nondigestible fractions also play an important role in modulating gut microbiome and the recognition of the importance of gut bacteria to obesity and metabolic dysfunction has increased in the past decade [16]. However, the effect of cooked whole black turtle beans on the gut microbiome remains unclear. The objectives of the present study were to investigate the anti-diabetic and anti-inflammatory effects of high fat (HF) diets enriched with cooked black turtle bean (HFB) on C57BL/6J mice and the possible mechanism, as well as the effect of HFB on gut microbiome.

## 2. Materials and Methods

### 2.1. Black Turtle Bean Preparation

Black turtle beans (Phaseolus vulgaris) were obtained from Bob’s Red Mill (Milwaukie, OR, USA). Soaked beans (50% hydration ratio) with water(*w*:*w* = 1:0.5)were cooked in a pressure cooker equipped with a pressure meter for 1 hr at 15 psi. Cooked beans were cooled and freeze dried and then ground using a food processor (Kitchen Aid, Benson Harbor, MI, USA).

### 2.2. Animals and Diets

C57BL/6J male mice were purchased from Jackson Laboratory (approximately 22 g, Sacramento, CA, USA). Mice were acclimated for 1 week and given water and chow diet (LabDiet #5001, PMI International, Redwood City, CA, USA) ad libitum. Mice were weighed and randomized into 3 groups of 8 mice each and fed high-fat (HF) diet (46% energy from fat), high-fat diet supplemented with 20% cooked black turtle bean (HFB) diet and low-fat (LF) diet (16% energy from fat). Macronutrients of the HF and HFB diets were adjusted to be equivalent in caloric content. Diet compositions are shown in Table 1. Body weights were recorded once a week and diet intake was monitored twice a week. This study was approved by the Institutional Animal Care and Use Committee, Western Regional Research Center, USDA, Albany, CA, USA (protocol No. 18-4, approval date 13 August 2018).

### 2.3. Blood and Tissue Sample Collection

Diets were removed from cages 12 h before sacrificing the mice at the end of the 6-week feeding study. Mice were anesthetized with isoflurane (Phoenix Pharmaceutical, St. Joseph, MO, USA) and blood was collected by cardiac puncture into EDTA pre-rinsed syringes. Plasma was obtained by centrifugation at 2000× *g* for 15 min at 4 °C and stored at −80 °C for further analysis. Liver and epididymal adipose were collected, weighed and frozen in liquid nitrogen immediately.

### 2.4. Blood Glucose, Plasma and Hepatic Lipid Analysis

OneTouch Ultrameter (LifeScan Inc., Milpitas, CA, USA) was used to determine the blood glucose concentration in the tail vein. Plasma lipoprotein cholesterol concentrations were determined by size-exclusion chromatography and postcolumn enzymatic reaction as described previously [17]. An enzyme colorimetric assay kit (Sekisui Diagnostics PEI Inc., Charlottetown, PE, Canada) was used to determine plasma triglyceride concentration per manufacture’s instruction. Liver lipids were extracted using a high temperature and pressure automated extractor (ASE, Dionex Corp., Sunnyvale, CA, USA) as described previously [18].

### 2.5. Glucose Tolerance Test (GTT) and Area under Curve (AUC)

Glucose tolerance tests were performed after 6 weeks of feeding. Mice were oral administrated 20% glucose solution (2 g/Kg body weight) after 5 h of fasting. Tail vein blood glucose concentration was acquired at 0, 15, 30, 60 and 120 min with OneTouch Ultrameter (LifeScan Inc., Milpitas, CA, USA). GTT curve was obtained by plotting glucose level versus time, and the measurement of glucose concentration over time was expressed as an integrated AUC.

### 2.6. Plasma Levels of Diabetes Related Biomarkers and Inflammatory Cytokines

Plasma ghrelin, GIP, GLP-1, insulin, leptin, resistin and glucagon levels were determined by mouse diabetes multiplex antibody assay kit and plasma inflammatory cytokines IL-2, IL-4, IL-5, IL-10, IL-12, GM-CSF, IFN-g and TNF-α were determined by mouse cytokine assay kit (Bio-Plex Pro Mouse Diabetes and Cytokine Assay 8-plex, Bio-Rad, Hercules, CA, USA) according to manufacturer’s instructions.

### 2.7. Homeostatic Model Assessment of Insulin Resistance (HOMA-IR) Index Calculation

Fasting glucose concentration and fasting plasma insulin level were used for HOMA-IR index calculation. HOMA-IR index was calculated according to the following equation [19].

$$\mathrm{HOMA}-\mathrm{IR}=\frac{\mathrm{fasting}\;\mathrm{plasma}\;\mathrm{insulin}\;\left(\mathrm{mU}/L\right)\times \mathrm{fasting}\;\mathrm{plasma}\;\mathrm{glucose}\;\left(\mathrm{mmol}/L\right)}{22.5}$$

### 2.8. Gut Microbiome Analysis

Mice were placed in paper cups and fecal pellets immediately collected, stored at -80°C. DNA from feces were extracted by Qiagen DNeasy PowerSoil kits (Qiagen, Valencia, CA, USA) following the standard protocol. The V3-V4 domains of the 16S rRNA were amplified using primers 319F/806R([TCGTCGGCAGCGTCAGATGTGTATAAGAGACAG(spacer)GTACTCCTACGGGAGGCAGCAGT] and [GTCTCGTGGGCTCGGAGATGTGTATAAGAGACAG(spacer)-CCGGACTACNVGGGTWTCTAAT] respectively) containing an Illumina tag sequence, a variable length spacer, linker sequence, and the 16S target sequence. Each sample was barcoded with an Illumina P5 adapter sequence, a unique 8 nucleotide (nt) barcode, a partial matching sequence of the forward primer, and reverse primers with an Illumina P7 adapter sequence, unique 8 nt barcode, and a partial matching sequence of the reverse adapter. The final product was quantified on a Qubit 4.0 instrument using the dsDNA Broad Range DNA kit (Invitrogen, Carlsbad, CA, USA) and individual amplicons were pooled in equal amounts. The pooled library was cleaned with Ampure XP beads (Beckman Coulter, Brea, CA, USA) and bands of interest were further isolated by gel electrophoresis (Sage Science, Beverly, MA, USA). The library was quantified via qPCR then sequenced with 300 base pair, dual end sequencing with an Illumina MiSeq at the Genome Center DNA Technologies Core, UC Davis.

The Raw FASTQ files and adapter trimmings were demultiplexed with dbcAmplicons version 0.8.5 (Available online: https://github.com/msettles/dbcAmplicons (accessed on 20 July 2021)). Forward and reverse unmerged reads were imported into QIIME2 version 2020.2 (Available online: https://qiime2.org (accessed on 20 July 2021)) and sequence variants were determined by utilizing the DADA2 analysis pipeline. Singletons and chimeras were removed as part of the quality filtering process and the remaining sequences were clustered into amplicon sequence variants (asv’s). The clustered sequences were then compared against the Silva 132 reference database which was used for taxonomic assignment meeting 99% identity. Shannon index was calculated and displayed with the R program (version 4.0.3, https://www.r-project.org/ (accessed on 18 January 2021)) through rarefactions to indicate alpha-diversity. Subsequently, operational taxonomic units (OTU) profiles of each sample were compared by the Bray-Curtis distance metric (using the vegan R package). The computed Bray-Curtis distances were used for Principal Coordinates Analysis (PCoA). The significance level for phylum difference was set at 0.05, while the OTU difference was set at 0.01.

### 2.9. RT-PCR Analysis

RNA was extracted from livers and adipose tissues by TRIzol and RNA purification kit (Invitrogen, Life Technologies, Carlsbad, CA, USA). All primers and probes for ddPCR were designed by Invitrogen (Invitrogen, Life Technologies, Carlsbad, CA, USA) as per Minimum Information for Publication of Quantitative Real-Time PCR Experiments (MIQE) guidelines [20]. Primer amplification efficiency was over 90% for every RT-PCR assay. cDNA was synthesized by GeneAmp RNA PCR kit (Applied Biosystems, Foster City, CA, USA). The synthesized cDNA was diluted 10 times with water, and 1 μL of diluted cDNA was applied in each RT-PCR using SYBR green super mix (Bio-Rad, Hercules, CA, USA) and analyzed by a Mx3000P instrument (Agilent, Cedar Creek, TX, USA). Cycle conditions were the same as our previous study [21]. PCR product sizes were analyzed by gel electrophoresis and no primer dimers were observed. Differences in mRNA expression in liver and epidydimal adipose tissues were calculated after normalization to β-actin or 36B4 mRNA expression.

### 2.10. Statistical Analysis

All data were presented as mean ± standard deviation. One-way ANOVA was performed to test the significant differences in groups means, followed by Tukey-Kramer HSD tests with 2016 SAS (Version 9.4, SAS Inc., Cary, NV, USA). Significance level was set at *p* < 0.05.

## 3. Results and Discussion

### 3.1. Metrics of Mice

To monitor the weight changes, energy intake, and other organ weights of mice fed different diet, the anthropometrics of mice fed HF, HFB, and LF diet were recorded and shown in Table 2. Body weight gain of mice fed HF diet was almost two times higher than mice on LF diet (Table 2). Total energy intake by mice on HFB was significantly lower than HF mice, but feed efficiency ratios were similar (Table 2, *p* > 0.05). Liver weight, adipose weight and fasting blood glucose of mice fed HFB diet were not significantly different from those on HF diet (Table 2). Reverri and coworkers reported decreased energy intake or appetite suppression in humans [22]. In Reverri’s study, they were fed meals containing either black beans or an external mix of fibers. While fiber is associated with satiety, the fiber contents of the three diets in the present study were adjusted to the same level. Reverri and coworkers [22] suggested that other components of whole black beans may have contributed to the satiety other than intrinsic fibers. Resistant starch is considered a type of dietary fiber and Garcia-Alonso et al. [23] reported that resistant starch in legumes significantly increased after cooking and cooling compared to the raw legumes. Freeze drying could also increase the level of resistant starch of cooked potato [24]. Da Silva et al. [25] reported that resistant starch could prolong satiety. These studies suggested that dietary fiber including resistant starch may be the cause of reduced energy intake by HFB diet. We adjusted the macronutrients in diet to the same level. However, the micronutrients which may had health promoting effect were not adjusted to the same level in the present study.

### 3.2. Plasma Lipoprotein, Triglyceride Levels and Hepatic Lipid Contents

**Figure 1:**
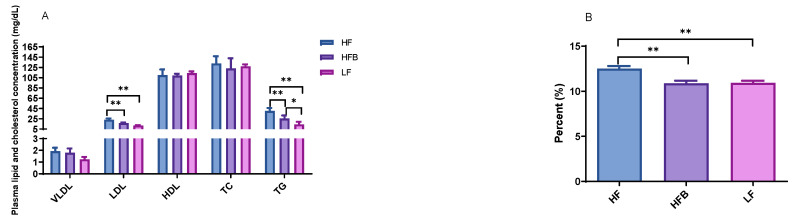
(**A**) Plasma lipid and cholesterol level in mice fed high-fat (HF) diet, high-fat diet containing 20% cooked black turtle bean (HFB) and low-fat (LF) diet for 6 weeks. (**B**) Hepatic total lipid content in mice fed with high fat (HF) diet, high-fat diet containing 20% cooked black turtle bean (HFB) and low-fat (LF) diet for 6 weeks. VLDL: very low-density lipoprotein; LDL: low-density lipoprotein; HDL: high-density lipoprotein; TC: total cholesterol; TG: triglyceride. * *p* < 0.05, ** *p* < 0.01 between groups.

Low-density lipoprotein (LDL) cholesterol and triglyceride (TG) concentrations of mice fed HFB diet were 28.0% and 36.6% lower than that of HF, respectively, than those of mice on HF diet (Figure 1A). Hepatic total lipid of the mice was lowered by HFB diet compared to HF diet (Figure 1B). The cholesterol lowering properties of soybeans, soy protein and soy isoflavones have been studied extensively, but reports of the effects of common dry beans on cholesterol metabolism in rodent models is scarce. With 9-month-old obese C57BL/6J mice fed HF diet containing 30% edible dry beans for 12 days, Zhu et al. [26] reported 17% lower total cholesterol compared to HF controls. LDL cholesterol was also 40% lower in bean fed mice. However, after fed HF diet supplemented with 30% edible dry beans for 7 weeks, the mice did not show any significant difference in total and LDL cholesterol in comparison with HF controls. In both the 12-day and 7-week studies, plasma TG was not different from control. Duane et al. [27] reported that legume consumption lowered serum LDL level and increased cholesterol saturation of bile, and they assumed that some components in legumes reduced the bile acid secretion and increased biliary cholesterol secretion independently. In our previous in vitro study, the fractionations of black turtle bean exhibited potent lipase inhibition activities [7]. To our knowledge, the present study is the first to report the significant reduction of plasma triglyceride and hepatic lipid content in C57BL/6J mice fed HFB diet compared to HF diet.

### 3.3. Glucose Tolerance Test (GTT) and Insulin Resistance

**Figure 2:**
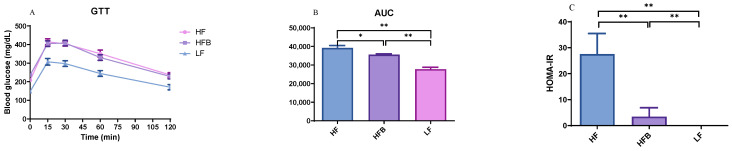
(**A**): Glucose tolerance test in mice fed high-fat (HF) diet, high-fat diet containing 20% cooked black turtle bean (HFB) and low-fat (LF) diet for 6 weeks; (**B**): Area under GTT curve after normalization; (**C**): HOMA-IR index of mice fed HF and HFB diets. * *p* < 0.05, ** *p* < 0.01 between groups. AUC: area under curve; GTT: glucose tolerance test; HOMA-IR: Homeostatic Model Assessment of Insulin Resistance (HOMA-IR) Index.

To understand glucose clearance from circulation and the insulin sensitivity of mice fed different diets, Glucose tolerance test (GTT) curve, the area under curve (AUC) and HOMA-IR index were investigated and shown in Figure 2A–C. Glucose clearance from circulation in mice fed HFB diet was manifested by 9% lower (*p* = 0.0005) AUC in comparison with HF. Insulin sensitivity was also improved as shown by 88% lower HOMA-IR index of mice fed HFB diet than those HF diet. HFB diet tended to lower blood glucose at 15, 60, and 120 min but did not reach statistical significance (*p* > 0.05). However, Tanaka et al. [28] reported lower peak glucose of GTT in mice on high-fat diets fats supplemented with boiled red kidney beans for 10 weeks. In the same study, the GTT of roasted red kidney beans was not different from high-fat controls. The researchers suggested that multiple synergistic effects of various components of the boiled beans were responsible.

Although the GTT AUC of mice fed HFB diet was lower than control (fasted for 5 h before testing), the fasting blood glucose concentration (FBG) just prior to sacrifice after 12 h fast was not different (Table 2). This might be due to the phenolic compounds in black turtle bean which possessed potent inhibitory activity on starch digesting enzymes [7]. Overall, the results indicated that black turtle beans ameliorated insulin resistance and preserved normal glucose metabolism even without affecting weight gain or abdominal adiposity.

### 3.4. Biomarkers Associated with Diabetes and Obesity

**Figure 3:**
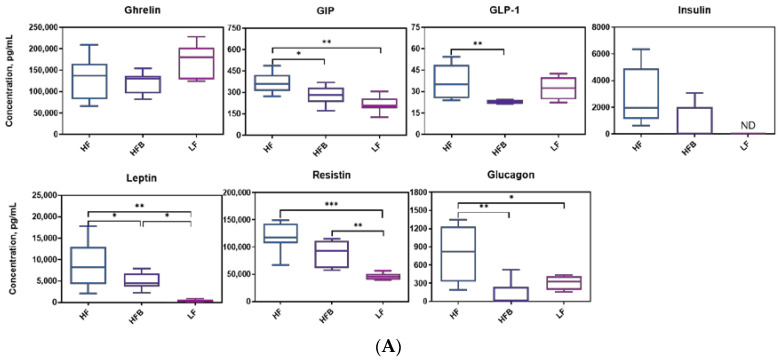

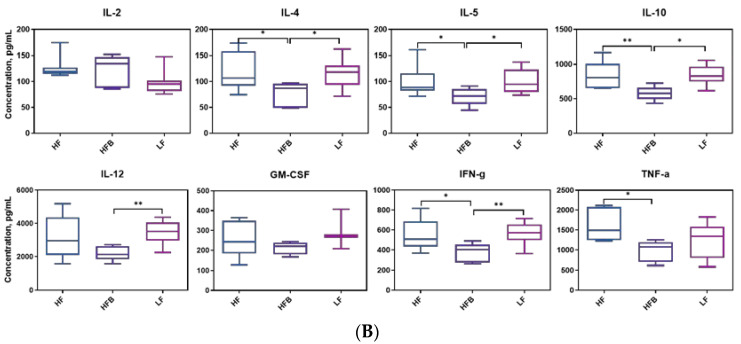
(**A**) Boxplot of plasma biomarkers concentrations related to diabetes and obesity, *n* = 8/group. ND = not detected. Top edge of the box, 75th percentile; bottom edge, 25th percentile; horizontal bar within box, median; top horizontal bar outside box, maximum concentration; bottom horizontal bar outside box, minimum concentration. * *p* < 0.05, ** *p* < 0.01, *** *p* < 0.001 between groups. (**B**) Concentrations of plasma inflammatory cytokines, *n* = 8/group. Top edge of the box, 75th percentile; bottom edge, 25th percentile; horizontal bar within box, median; top horizontal bar outside box, maximum concentration; bottom horizontal bar outside box, minimum concentration. * *p* < 0.05, ** *p* < 0.01 between groups.

Since HOMA-IR of mice were diminished by HFB diet, the levels of circulating proteins involved in the regulation of glucose metabolism were measured. Concentration of diabetes and obesity related biomarkers are shown in Figure 3A. Mice fed HFB diet had 23.4%, 21.6%, 43.2% and 52.1% lower GIP, GLP-1, leptin and glucagon concentrations than those on HF diet, respectively. Plasma ghrelin, insulin and resistin levels of the HFB and HF diets were not different. However, in the LF and HFB groups, insulin was below the detection limit in 8 and 3 samples, respectively, whereas, of the 8 samples analyzed, all mice in the HF group had detectable levels of insulin. Concentration of diabetes and obesity related biomarkers are shown in Figure 3A. Mice fed HFB diet had 23.4%, 21.6%, 43.2% and 52.1% lower GIP, GLP-1, leptin and glucagon concentrations than those on HF diet, respectively. Plasma ghrelin, insulin and resistin levels of the HFB and HF diets were not different. However, in the LF and HFB groups, insulin was below the detection limit in 8 and 3 samples, respectively, whereas, of the 8 samples analyzed, all mice in the HF group had detectable levels of insulin.

**Figure 4:**
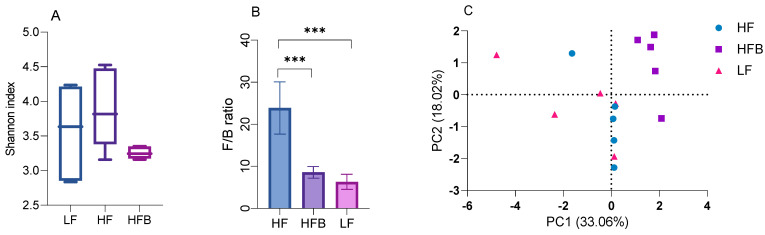

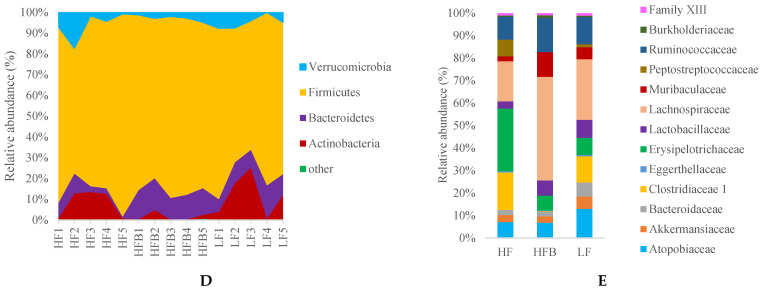
Box plots of (**A**) Shannon Index and (**B**) the ratio of Firmicutes to Bacteroidetes in mice fed each diet for 6 weeks, and (**C**) principal co-ordinate analysis (PCoA) plot of cecal microbial community data (**D**) the relative abundance of cecal microbiota at the Phylum level, and (**E**) the relative abundance of cecal microbiota at the Family level. *** *p* < 0.001 between groups.

Ghrelin is an orexigenic hormone secreted mainly by the stomach preprandially and is responsible for regulating appetite and energy hemostasis. Ghrelin levels in this study were not different among LF, HF and HFB fed mice. However, energy intakes of mice fed LF and HFB diet were significantly lower than HF diet (Table 2). Ghrelin levels are higher before meals and lower between meals. In the current study mice were sacrificed after 12 h fasting, and therefore high ghrelin levels in mice were expected. Gastric inhibitory polypeptide (GIP) is a glucose dependent insulinotropic peptide, and responsible for stimulating insulin secretion. Nasteska et al. [29] generated a GIP knockout mouse model and reported that the knockout had similar GTT response to the wild type on HF diets but reduced obesity and insulin resistance through higher fat oxidation. Increased fat oxidation and elevated adiponectin levels were found in GIP-receptor null mice [30]. The GIP-receptor knockout mice also had higher adiponectin mRNA expression. In the current study we also found increased adiponectin (Figure 4B) expression, lower GIP, and lower insulin resistance in HFB fed mice. Leptin is a hormone secreted by adipocytes that regulates energy balance via inhibiting hunger. Leptin level in mice fed HFB diet was about 50% lower than leptin in mice on the HF diet. Energy intake of mice fed HFB was also significantly lower than that of the mice on HF diet. Higher energy intake of the mice on HF diet may be due to decreased sensitivity to leptin or leptin resistance. C57BL/6J mice fed a 45% fat diet became peripherally leptin resistant after 16 d [31]. Although peripheral leptin resistance occurred after 16 d, central leptin sensitivity (by intracerebroventricular infusion) was not affected [31]. Leptin levels may also be modulated by the phenolic compounds in black turtle bean. Fasting glucagon level of mice fed HF diet was 52.1% higher than that on HFB diet and similar to levels on LF diet. In humans high fasting glucagon level was reported to be correlated with the insulin resistance [32].

### 3.5. Plasma Levels of Inflammatory Cytokines

Biomarkers involved in the regulation of glucose metabolism were affected by HFB diet. We therefore tested the inflammatory cytokines in plasma. Inflammatory cytokines interact with specific receptors on target cells to mediate inflammation. Plasma inflammatory cytokine levels are shown in Figure 3B. Although there were significant differences in body weight, liver and adipose weight, fasting blood glucose and HOMA-IR between mice fed the HF and LF diets, there were no differences in 7 cytokines (except TNF-α) level between HF and LF treatments.

The lack of significance between the HF and LF treatments was partly due to the higher variance in the LF diet group and perhaps insufficient length of the feeding phase to generate an immune response. The latter is suggested by a study by Kobori et al. [33] who found that TNF-α levels were 2.4, 4.7 and 7.3 times higher in C57BL/6J mice fed HF diet for 4, 8, and 20 weeks, respectively, compared to AIN-93G (LF) control. Other studies have shown differences after 8 weeks [34]. Although there were no differences in cytokines between HF and LF feeding, there were differences between HF and HFB diet groups. Plasma IL-4, IL-5, IL-10, IL-12, IFN-g and TNF-α concentrations of mice fed HFB diet were 34.3%, 30.82%, 31.30%, 31.34%, 30.19% and 30.81% less than those on HF diet. IL-10 is an anti-inflammatory cytokine that responsible for inhibiting TNF-α, IL-12 and IFN-γ secretion [35]. IL-4 was higher in spleens from mice fed high-fat diets cultured in phytohaemagglutin (PHA) compared to controls on LF diet [36]. Black turtle beans contain significant amounts of PHA but they are detoxified by cooking. In this study the body weights of mice on the HFB diet were lower than HF diet but not significantly. Higher intake of animal proteins were positively associated with inflammation in obese individuals and no correlation has been observed between plant protein intake and inflammation in obese subjects [36]. However, the mechanism of the effect of animal or plant based protein on inflammatory cytokines remains unclear. Food is a complicated system. Typically there are more than one component attributed to a health promoting effect. There are also some synergistic effects and antagonistic effects in the same foods. To better explain the positive effect of cooked black turtle bean on health promotion field, the separation, purification and functional evaluation of its components will be conducted in the future.

### 3.6. Gut Microbiome Analysis

In order to understand the effects of the HFB diet on the gut microbiome, fecal samples of mice fed the different diets were analyzed. The Shannon index (Figure 4A) of the gut microbiota in HFQ group is similar to that of the HF and LF groups, suggesting that cooked black turtle bean supplementation does not affect the diversity of gut microbiota. The ratio of Firmicutes/Bacteroidetes (F/B) was also decreased 64.1% by HFB diet compared to HF diet (Figure 4B). The reduction of F/B ratio in early studies suggested that Bacteroidetes were responsible for favorable metabolic changes [37]. Principal coordinate analysis (PCoA) (Figure 4C), PC1 (33.06%) separates mice fed HFB diet, and PC2 (18.02%) roughly separates the mice fed HF and LF diets. At the phylum level, relative abundances of Bacteroidetes and Firmicutes were increased (64.3%) and decreased (not significantly) by HFB diet compared to HF diet (Figure 4D). At the family level, there were differences in the relative bacteria composition in feces of mice fed HF, HFB and LF diets (Figure 4E). HFB diet significantly induced the enrichment of Lachnospiraceae and Muribaculaceae compared to HF and LF diet. Rats fed HF diets supplemented with pea fiber had improved glucose tolerance and higher levels of Lachnospiracease compared to controls [38]. In a subgroup of lean mice on HF diets, Muribaculaceae was associated with resistance to weight gain compared to the fatter mice [39], suggested that mice fed HFB diet is more resistance to obesity than that of mice fed HF diet alone. In contrast, Peptostreptococcaceae was not detected and Erysipelotrichaceae was reduced in mice fed HFB diet. These groups were also decreased in a study of mice that were fed HF diets and orally administered a tea extract [40]. The relative abundance of Clostridiaceae 1 was also significantly reduced by HFB diet compared to LF and HF diet. At the genus level, the relative abundance of *Blautia*, *Clostridium sensu stricto 1*, *Erysipelatoclostridium*, *Romboutsia* and *Turicibacter* in mice fed HFB diet was significantly reduced compared to HF diet. B6 mice fed a HF diet supplemented with resistant starch from tartary buckwheat also reduced *Erysipelotoclostridium* and *Turicibacter* but increased *Blautia* compared to mice fed HF with a resistant starch from corn [41]. The high resistant starch content of legumes may have contributed to the changes in these genera. In contrast, relative abundance of *Lachnospiraceae NK4A136 group* and *Ruminococcus 1* was enriched in feces of mice fed HFB diet compared to HF diet. HF diets have been reported to reduce *Lachnospiraceae NK4A136 group*, a SCF producing species [42] while HF diets supplemented with the dietary fiber, inulin, had lower *Ruminococcus 1* [43]. These studies suggest that the dietary fiber or resistant starch content of HFB diets facilitated the growth of short chain fatty acid producing bacteria.

At the phylum level, Actinobacteria, Bacteroidetes and Firmicutes were the dominant bacterial phylum. The reduction of Firmicutes and significant increase in Bacteroidetes resulted in a significantly lower F/B ratio in mice fed HFB and LF diet compared to HF diet. Sheflin et al. [44] reported that the significant reduction of F/B ratio in colorectal cancer survivors consuming rice bran and cooked navy bean for 28 days, but the reduction in navy bean diet did not reach significance due to the large variation. Reduction of F/B ratio in obese subjects is usually considered to be correlated with weight loss [45]. However, mice fed HFB diet gained 14.9% less weight compared to those on HF diet but was not statistically significant.

At the family level, the relative abundance of Peptostreptococcaceae, Clostridiaceae 1 and Erysipelotrichaceae was reduced by HFB diet compared to HF diet. Monk et al. [10] reported that cranberry bean consumption reduced the relative abundance of Peptostreptococcaceae and Clostridiaceae. Peptostreptococcaceae and Clostridiaceae belong to Firmicutes phylum, Clostridia class. Martinez et al. [46] reported the reduction of Erysipelotrichaceae in hypercholesterolemia hamster treated with sorghum extract. Erysipelotrichaceae was reported to be positively correlated with host cholesterol metabolites [47]. The reduction of plasma LDL, TG levels and relative abundance of Erysipelotrichaceae observed in the present study supports the association between Erysipelotrichaceae and host lipid metabolism. At the genus level, abundance of *Blautia*, *Clostridium sensu stricto 1*, intestinal inflammation bacteria *Erysipelatoclostridium* [48], *Romboutsia* and *Turicibacter* genera was significantly decreased by HFB diet compared to HF diet, where *Blautia* was inversely correlated with visceral fat [49].

### 3.7. RT-PCR Analysis

**Figure 5:**
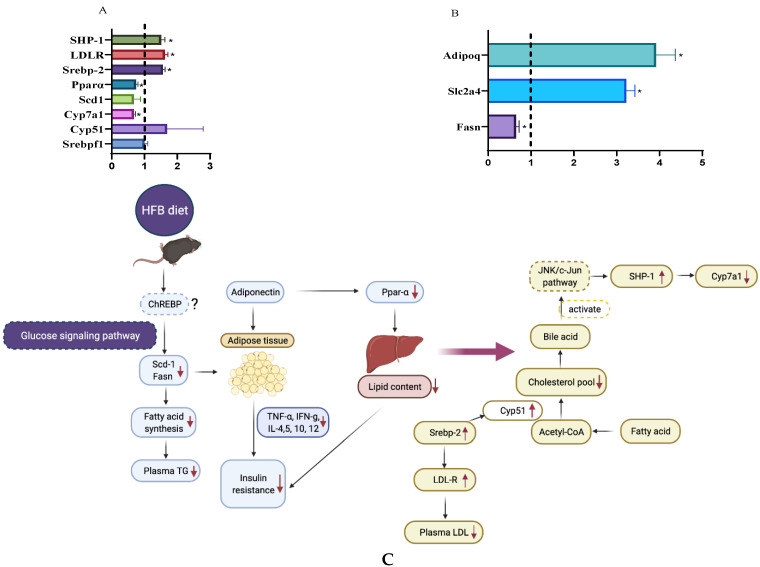
Relative hepatic gene expression of *Srebf1*, *Cyp51*, *Cyp7a1*, *Scd1*, *Ppara*, *Screbp-2*, and *LDL-R* in mice fed HFQ diet compared to HF diet (**A**). Relative expression of *Fasn*, *Slc2a4* and *Adipoq* genes in epididymal adipose tissue of mice fed HFQ diet compared to HF diet (**B**). Data are expressed as mean ± SEs, and *n* = 4/group. Differences in mRNA expression for livers and adipose were calculated after normalizing to 35B4 mRNA expression. Asterisk indicates significant difference (*p* < 0.05). Dotted line (x = 1) represents the expression of control gene. (**C**) Proposed mechanism responsible for ameliorating insulin resistance in mice fed HF diet supplemented with cooked black turtle beans, glucose signaling pathway, JNK/c-Jun pathway and ChREBP (dashes outline) were extrapolated from literature [50,51].

Because GTT AUC (area of curve), plasma TG and LDL were lowered by the HFB diet, we compared the expression of selected genes related to fat and glucose metabolism. Relative gene expression in liver and adipose tissues is shown in Figure 5A,B.

Expressions of hepatic genes involved in cholesterol synthesis (*Cyp51*, *Srebp-2*, *LDL-R*) and bile acid synthesis (*Cyp7a1*), were 1.69, 1.56, 1.62 and 0.67 times than those derived from mice on the HF diet, respectively. Contrary to our results, Tanaka and coworkers reported that roasted red kidney beans tended to increase the expression of *HMGCoAR*, a gene in the cholesterol synthesis and significantly increased *Cyp7a1* [28]. Fatty acid metabolism related genes in liver and adipose, *Scd1*, *Pparα* and *Fasn* were reduced by 33, 26 and 35%, respectively. Glucose and fatty acid metabolism related gene, *Adipoq*, coding for the adipokine, adiponectin, was 2.91 folds greater expressed than HF diet. Insulin-regulated glucose transporter 4 (*Slc2a4*) was expressed 2.22 folds more when HFB diet was administered. Although adipose weight and body weight gain were not significantly different between HFB and HF (Table 2), the fatty acid metabolism related genes expression results support lower fatty acid synthesis and lipid desaturation for fat storage. *Scd1* and *Fasn* are the target genes of *Srebpf1* [52]. However, the reduction of *Scd1* and *Fasn* were not accompanied by decreasing of *Srebpf1*, suggesting that other pathways exist to regulate the expression of *Scd1* and *Fasn*. Postic et al. [51] reported that TG synthesis in liver was regulated by carbohydrate-responsive element–binding protein (ChREBP) and sterol-regulatory-element-binding protein 1c (Srebpf1). ChREBP was involved in glucose signaling pathway, and Srebpf1 was related to insulin signaling pathway [51]. Therefore, we hypothesize that the HFB diet decreased the lipogenesis (*Fasn*) and desaturation/elongation of fat (*Scd1*) and then subsequently reduced plasma TG in mice via the glucose signaling pathway. While LDL-cholesterol was decreased in mice fed HFB diet, the increase in gene expression of *Cyp51* was significant but slight. However, *Cyp7a1* was down-regulated by HFB diet, and bile acid might down-regulate *Cyp7a1* gene through activation of JNK/c-Jun pathway [52]. The expression of Slc2a4 and Adipoq were upregulated by HFB diet and the decreased expression (mice on HF diet group) of *Slc2a4* and *Adipoq* (coding for adiponectin) are associated with insulin resistance [53,54]. These results support the lowered insulin resistance by HFB is related to insulin and other blood biomarkers. Proposed mechanism of responsible for ameliorating insulin resistance in mice fed HF diet supplemented with cooked black turtle beans are shown in Figure 5C.

The present study is the first to report the significant reduction of plasma triglyceride and hepatic lipid content in C57BL/6J mice fed high fat diet supplemented with cooked black turtle beans compared to high fat diet alone. However, the resistant starch level would increase in cooked beans and might affect the digestion rate and not adjusted to the same level in the present study. And the tran-C57BL/6J mice were used in the present study, results of the current study need further clinical trials to confirm.

## 4. Conclusions

In summary, the present study demonstrated that cooked black turtle bean supplementation in a HF diet had health promoting effects in terms of lipid and glucose metabolism. HFB diet ameliorated insulin resistance and lowered plasma LDL in mice fed HF diet through glucose signaling pathway and JNK/c-Jun pathway. Diabetes related biomarkers: GIP, GLP-1, leptin, glucagon and inflammatory cytokines: IL-4, IL-5, IL-10, IL-12, IFN-g and TNF-α were significantly affected by HFB diet compared to HF diet. Cooked black turtle bean supplementation did not affect the diversity of gut microbiota compared to HF alone. Firmicutes/Bacteroidetes (F/B) ratio was decreased 64.1% by HFB diet. HFB diet significantly induced the enrichment of Lachnospiraceae and Muribaculaceae compared to HF and LF diet.

## Figures and Tables

**Table 1:** Diet compositions (g).

Ingredient	High-Fat Diet (HF)	High-Fat Diet with Beans (HFB)	Low-Fat Diet (LF)
Lard fat	225.0	225.0	63.0
Soybean oil	25.0	23.2	7.0
Cholesterol	0.8	0.8	0.8
Cellulose	50.0	17.0	50.0
20% beans	-	200.0	-
Casein	200.0	157.5	200.0
Corn Starch	148.2	25.5	528.2
Sucrose	300.0	300.0	100.0
Cystine	3.0	3.0	3.0
Choline Bitartrate	3.0	3.0	3.0
Mineral Mix	35.0	35.0	35.0
Vitamin Mix	10.0	10.0	10.0
Total weight	1000.0	1000.0	1000.0
Calories/Kg	4850.0	4839.0	3950.0

**Table 2:** Anthropometrics in mice fed different diets for 6 weeks.

	HF	HFB	LF
Body weight gain (g)	12.41 ± 0.82a	10.56 ± 0.85a	4.40 ± 0.28b
Total energy intake (Kcal)	707.00 ± 37.8a	615.00 ± 13.2b	587.00 ± 20.2b
Feed efficiency ratio (g gain/g feed)	0.09 ± 0.01a	0.08 ± 0.01a	0.03 ± 0.01b
Feed efficiency ratio (g gain/Kcal feed)	0.02 ± 0.002a	0.02 ± 0.001a	0.01 ± 0.001b
Liver weight (g)	1.02 ± 0.03a	1.03 ± 0.03a	0.91 ± 0.05b
Liver/body weight	0.03 ± 0.0003b	0.03 ± 0.0008ab	0.03 ± 0.0011a
Adipose weight (g)	0.96 ± 0.07a	0.80 ± 0.08a	0.31 ± 0.03b
Adipose/body weight	0.03 ± 0.0016a	0.02 ± 0.0021a	0.01 ± 0.0008b
Fasting blood glucose (mg/dL)	157.88 ± 11.21a	167.75 ± 9.76a	122.88 ± 8.88b

Values are means ± SEM, *n* = 8. Different letters within the same row indicate significant difference at *p* < 0.05 level. HF: High-fat control diet (46% Kcal from fat, 16.5% Kcal from protein, and 37.5% Kcal from carbohydrate); HFB: 20% cooked black turtle beans in high-fat diet; LF: Low-fat control diet (16% Kcal from fat, 20% Kcal from protein, and 64% Kcal from carbohydrate).

## Data Availability

Data is contained within the article.

## References

[1] Akibode C.S., Maredia M.K. (2012). Global and Regional Trends in Production, Trade and Consumption of Food Legume Crops.

[2] Minor T., Bond J.K. (2018). Vegetables and Pulses Outlook.

[3] Havemeier S., Erickson J., Slavin J. (2017). Dietary guidance for pulses: The challenge and opportunity to be part of both the vegetable and protein food groups. Ann. N. Y. Acad. Sci..

[4] Singh B., Singh J.P., Kaur A., Singh N. (2017). Phenolic composition and antioxidant potential of grain legume seeds: A review. Food Res. Int..

[5] Ganesan K., Xu B. (2017). Polyphenol-rich dry common beans (*Phaseolus vulgaris* L.) and their health benefits. Int. J. Mol. Sci..

[6] Xu B.J., Chang S. (2007). A comparative study on phenolic profiles and antioxidant activities of legumes as affected by extraction solvents. J. Food Sci..

[7] Tan Y., Chang S.K., Zhang Y. (2017). Comparison of α-amylase, α-glucosidase and lipase inhibitory activity of the phenolic substances in two black legumes of different genera. Food Chem..

[8] Tan Y., Chang S.K. (2017). Digestive enzyme inhibition activity of the phenolic substances in selected fruits, vegetables and tea as compared to black legumes. J. Funct. Foods.

[9] Hernández-Saavedra D., Mendoza-Sánchez M., Hernández-Montiel H.L., Guzmán-Maldonado H.S., Loarca-Piña G.F., Salgado L.M., Reynoso-Camacho R. (2013). Cooked common beans (*Phaseolus vulgaris*) protect against β-cell damage in streptozotocin-induced diabetic rats. Plant Foods Hum. Nutr..

[10] Monk J.M., Lepp D., Zhang C.P., Wu W., Zarepoor L., Lu J.T., Pauls K.P., Tsao R., Wood G.A., Robinson L.E. (2016). Diets enriched with cranberry beans alter the microbiota and mitigate colitis severity and associated inflammation. J. Nutr. Biochem..

[11] Liu Z., Bruins M.E., Ni L., Vincken J.-P. (2018). Green and black tea phenolics: Bioavailability, transformation by colonic microbiota, and modulation of colonic microbiota. J. Agric. Food Chem..

[12] Chen Y., Chang S.K.C., Zhang Y., Hsu C.-Y., Nannapaneni R. (2020). Gut microbiota and short chain fatty acid composition as affected by legume type and processing methods as assessed by simulated in vitro digestion assays. Food Chem..

[13] Bravo L., Siddhuraju P., Saura-Calixto F. (1998). Effect of various processing methods on the in vitro starch digestibility and resistant starch content of indian pulses. J. Agric. Food Chem..

[14] Zhang Z., Lanza E., Kris-Etherton P.M., Colburn N.H., Bagshaw D., Rovine M.J., Ulbrecht J.S., Bobe G., Chapkin R.S., Hartman T.J. (2010). A high legume low glycemic index diet improves serum lipid profiles in men. Lipids.

[15] Carbonaro M., Maselli P., Nucara A. (2015). Structural aspects of legume proteins and nutraceutical properties. Food Res. Int..

[16] Lovegrove A., Edwards C., De Noni I., Patel H., El S., Grassby T., Zielke C., Ulmius M., Nilsson L., Butterworth P. (2017). Role of polysaccharides in food, digestion, and health. Crit. Rev. Food Sci. Nutr..

[17] Kim H., Bartley G.E., Arvik T., Lipson R., Nah S.-Y., Seo K., Yokoyama W. (2014). Dietary supplementation of chardonnay grape seed flour reduces plasma cholesterol concentration, hepatic steatosis, and abdominal fat content in high-fat diet-induced obese hamsters. J. Agric. Food Chem..

[18] Shao D., Bartley G.E., Yokoyama W., Pan Z., Zhang H., Zhang A. (2013). Plasma and hepatic cholesterol-lowering effects of tomato pomace, tomato seed oil and defatted tomato seed in hamsters fed with high-fat diets. Food Chem..

[19] Bowe J.E., Franklin Z.J., Hauge-Evans A.C., King A.J., Persaud S.J., Jones P.M. (2014). Metabolic phenotyping guidelines: Assessing glucose homeostasis in rodent models. J. Endocrinol..

[20] Bustin S.A., Benes V., Garson J.A., Hellemans J., Huggett J., Kubista M., Mueller R., Nolan T., Pfaffl M.W., Shipley G.L. (2009). The Miqe Guidelines: M Inimum i Nformation for Publication of q Uantitative Real-Time pcr e Xperiments.

[21] Kim H., Kim D.-H., Seo K.-H., Chon J.-W., Nah S.-Y., Bartley G.E., Arvik T., Lipson R., Yokoyama W. (2015). Modulation of the intestinal microbiota is associated with lower plasma cholesterol and weight gain in hamsters fed chardonnay grape seed flour. J. Agric. Food Chem..

[22] Reverri E.J., Randolph J.M., Kappagoda C.T., Park E., Edirisinghe I., Burton-Freeman B.M. (2017). Assessing beans as a source of intrinsic fiber on satiety in men and women with metabolic syndrome. Appetite.

[23] García-Alonso A., Goni I., Saura-Calixto F. (1998). Resistant starch and potential glycaemic index of raw and cooked legumes (lentils, chickpeas and beans). Z. Lebensm. Forsch. A.

[24] Mishra S., Monro J., Hedderley D. (2008). Effect of processing on slowly digestible starch and resistant starch in potato. Starch-Stärke.

[25] Da Silva C.S., Haenen D., Koopmans S.J., Hooiveld G.J., Bosch G., Bolhuis J.E., Kemp B., Müller M., Gerrits W.J. (2014). Effects of resistant starch on behaviour, satiety-related hormones and metabolites in growing pigs. Animal.

[26] Zhu Z., Jiang W., Thompson H.J. (2012). Edible dry bean consumption (phaseolus vulgaris l.) modulates cardiovascular risk factors and diet-induced obesity in rats and mice. Br. J. Nutr..

[27] Duane W. (1997). Effects of legume consumption on serum cholesterol, biliary lipids, and sterol metabolism in humans. J. Lipid Res..

[28] Tanaka M., Honda Y., Miwa S., Akahori R., Matsumoto K. (2019). Comparison of the effects of roasted and boiled red kidney beans (*Phaseolus vulgaris* L.) on glucose/lipid metabolism and intestinal immunity in a high-fat diet-induced murine obesity model. J. Food Sci..

[29] Nasteska D., Harada N., Suzuki K., Yamane S., Hamasaki A., Joo E., Iwasaki K., Shibue K., Harada T., Inagaki N. (2014). Chronic reduction of gip secretion alleviates obesity and insulin resistance under high-fat diet conditions. Diabetes.

[30] Naitoh R., Miyawaki K., Harada N., Mizunoya W., Toyoda K., Fushiki T., Yamada Y., Seino Y., Inagaki N. (2008). Inhibition of gip signaling modulates adiponectin levels under high-fat diet in mice. Biochem. Biophys. Res. Commun..

[31] Van Heek M., Compton D.S., France C.F., Tedesco R.P., Fawzi A.B., Graziano M.P., Sybertz E.J., Strader C.D., Davis H.R. (1997). Diet-induced obese mice develop peripheral, but not central, resistance to leptin. J. Clin. Investig..

[32] Ferrannini E., Muscelli E., Natali A., Gabriel R., Mitrakou A., Flyvbjerg A., Golay A., Hojlund K. (2007). Association of fasting glucagon and proinsulin concentrations with insulin resistance. Diabetologia.

[33] Kobori M., Masumoto S., Akimoto Y., Oike H. (2011). Chronic dietary intake of quercetin alleviates hepatic fat accumulation associated with consumption of a western-style diet in c57/bl6j mice. Mol. Nutr. Food Res..

[34] Peng J., Jia Y., Hu T., Du J., Wang Y., Cheng B., Li K. (2019). Gc-(4 → 8)-gcg, a proanthocyanidin dimer from camellia ptilophylla, modulates obesity and adipose tissue inflammation in high-fat diet induced obese mice. Mol. Nutr. Food Res..

[35] Anitescu M., Chace J.H., Tuetken R., YI A.-K., Berg D.J., Krieg A.M., Cowdery J.S. (1997). Interleukin-10 functions in vitro and in vivo to inhibit bacterial DNA-induced secretion of interleukin-12. J. Interferon Cytokine Res..

[36] Mito N., Hosoda T., Kato C., Sato K. (2000). Change of cytokine balance in diet-induced obese mice. Metabolism.

[37] Lopez-Legarrea P., de la Iglesia R., Abete I., Navas-Carretero S., Martinez J.A., Zulet M.A. (2014). The protein type within a hypocaloric diet affects obesity-related inflammation: The resmena project. Nutrition.

[38] Ley R.E., Turnbaugh P.J., Klein S., Gordon J.I. (2006). Human gut microbes associated with obesity. Nature.

[39] Hashemi Z., Fouhse J., Im H.S., Chan C.B., Willing B.P. (2017). Dietary pea fiber supplementation improves glycemia and induces changes in the composition of gut microbiota, serum short chain fatty acid profile and expression of mucins in glucose intolerant rats. Nutrients.

[40] Cao W., Chin Y., Chen X., Mi Y., Xue C., Wang Y., Tang Q. (2020). The role of gut microbiota in the resistance to obesity in mice fed a high fat diet. Int. J. Food Sci. Nutr..

[41] Lu X., Liu J., Zhang N., Fu Y., Zhang Z., Li Y., Wang W., Li Y., Shen P., Cao Y. (2019). Ripened pu-erh tea extract protects mice from obesity by modulating gut microbiota composition. J. Agric. Food Chem..

[42] Zhou Y., Wei Y., Yan B., Zhao S., Zhou X. (2020). Regulation of tartary buckwheat-resistant starch on intestinal microflora in mice fed with high-fat diet. Food Sci. Nutr..

[43] Peng L., Zhang Q., Zhang Y., Yao Z., Song P., Wei L., Zhao G., Yan Z. (2020). Effect of tartary buckwheat, rutin, and quercetin on lipid metabolism in rats during high dietary fat intake. Food Sci. Nutr..

[44] Zhang Q., Li C., Niu X., Zhang Z., Li F., Li F. (2019). The effects of milk replacer allowance and weaning age on the performance, nutrients digestibility, and ruminal microbiota communities of lambs. Anim. Feed. Sci. Technol..

[45] Sheflin A.M., Borresen E.C., Kirkwood J.S., Boot C.M., Whitney A.K., Lu S., Brown R.J., Broeckling C.D., Ryan E.P., Weir T.L. (2017). Dietary supplementation with rice bran or navy bean alters gut bacterial metabolism in colorectal cancer survivors. Mol. Nutr. Food Res..

[46] Duca F.A., Sakar Y., Lepage P., Devime F., Langelier B., Doré J., Covasa M. (2014). Replication of obesity and associated signaling pathways through transfer of microbiota from obese-prone rats. Diabetes.

[47] Martínez I., Wallace G., Zhang C., Legge R., Benson A.K., Carr T.P., Moriyama E.N., Walter J. (2009). Diet-induced metabolic improvements in a hamster model of hypercholesterolemia are strongly linked to alterations of the gut microbiota. Appl. Environ. Microbiol..

[48] Martínez I., Perdicaro D.J., Brown A.W., Hammons S., Carden T.J., Carr T.P., Eskridge K.M., Walter J. (2013). Diet-induced alterations of host cholesterol metabolism are likely to affect the gut microbiota composition in hamsters. Appl. Environ. Microbiol..

[49] Wang S., Lv Z., Zhao W., Wang L., He N. (2020). Collagen peptide from walleye pollock skin attenuated obesity and modulated gut microbiota in high-fat diet-fed mice. J. Funct. Foods.

[50] Bartley G.E., Yokoyama W., Young S.A., Anderson W.H., Hung S.-C., Albers D.R., Langhorst M.L., Kim H. (2010). Hypocholesterolemic effects of hydroxypropyl methylcellulose are mediated by altered gene expression in hepatic bile and cholesterol pathways of male hamsters. J. Nutr..

[51] Postic C., Girard J. (2008). Contribution of de novo fatty acid synthesis to hepatic steatosis and insulin resistance: Lessons from genetically engineered mice. J. Clin. Investig..

[52] Ozato N., Saito S., Yamaguchi T., Katashima M., Tokuda I., Sawada K., Katsuragi Y., Kakuta M., Imoto S., Ihara K. (2019). Blautia genus associated with visceral fat accumulation in adults 20–76 years of age. NPJ Biofilms Microbiomes.

[53] Gupta S., Stravitz R.T., Dent P., Hylemon P.B. (2001). Down-regulation of cholesterol 7α-hydroxylase (cyp7a1) gene expression by bile acids in primary rat hepatocytes is mediated by the c-jun n-terminal kinase pathway. J. Biol. Chem..

[54] Abel E.D., Peroni O., Kim J.K., Kim Y.-B., Boss O., Hadro E., Minnemann T., Shulman G.I., Kahn B.B. (2001). Adipose-selective targeting of the glut4 gene impairs insulin action in muscle and liver. Nature.

